# *Drosophila* cells use nanotube-like structures to transfer dsRNA and RNAi machinery between cells

**DOI:** 10.1038/srep27085

**Published:** 2016-06-03

**Authors:** Margot Karlikow, Bertsy Goic, Vanesa Mongelli, Audrey Salles, Christine Schmitt, Isabelle Bonne, Chiara Zurzolo, Maria-Carla Saleh

**Affiliations:** 1 Institut Pasteur, Viruses and RNA interference, CNRS URM 3569, 75724 Paris Cedex 15, France; 2 Sorbonne Universités, UPMC Université Paris VI, IFD, 4 Place Jussieu 75252 Paris Cedex 05, France; 3 Institut Pasteur, Imagopole, Citech, 75724 Paris Cedex 15, France; 4 Institut Pasteur, Platform of Ultra-structural microscopy, 75724 Paris Cedex 15, France; 5 Institut Pasteur, Membrane traffic and pathogenesis, 75724 Paris Cedex 15, France

## Abstract

Tunnelling nanotubes and cytonemes function as highways for the transport of organelles, cytosolic and membrane-bound molecules, and pathogens between cells. During viral infection in the model organism *Drosophila melanogaster*, a systemic RNAi antiviral response is established presumably through the transport of a silencing signal from one cell to another *via* an unknown mechanism. Because of their role in cell-cell communication, we investigated whether nanotube-like structures could be a mediator of the silencing signal. Here, we describe for the first time in the context of a viral infection the presence of nanotube-like structures in different *Drosophila* cell types. These tubules, made of actin and tubulin, were associated with components of the RNAi machinery, including Argonaute 2, double-stranded RNA, and CG4572. Moreover, they were more abundant during viral, but not bacterial, infection. Super resolution structured illumination microscopy showed that Argonaute 2 and tubulin reside inside the tubules. We propose that nanotube-like structures are one of the mechanisms by which Argonaute 2, as part of the antiviral RNAi machinery, is transported between infected and non-infected cells to trigger systemic antiviral immunity in *Drosophila*.

To establish systemic immunity and protect both the site of initial infection and the entire organism, all multicellular species have developed sophisticated ways to communicate immune signals. These signals must be disseminated between cells locally and throughout the organism to avoid pathogen propagation and establishment of infection. Not long ago, it was proposed that mammalian immune cells (such as dendritic cells and macrophages) transmit signals to distant cells through a network of physically connected tunneling nanotubes (TNT)^1^,^2^,^3^,^4^. TNTs were first described in rat neuro-derived cells^5^, and since then in a wide variety of mammalian cells where they act as a transport route for cytosolic and membrane-bound molecules, organelles, and pathogens such as HIV and prions^6^,^7^,^8^,^9^,^10^,^11^. Analogous structures are widely observed. In higher plants, plasmodesmata (a structure composed of desmotubules)^12^,^13^ are continuously lined by the plasma membrane allowing the transport of molecules such as nutrients, hormones, regulatory proteins, and RNA from one cell to another^14^,^15^,^16^. In bacteria, nanotubes bridge neighbouring cells for exchange of molecules within and between species^17^. Filamentous connections, that resemble nanotubes, link gametes during malarial parasite reproduction in the mosquito midgut^18^. In *Drosophila*, cytonemes in the wing imaginal disc are a type of filopodium in which morphogen signalling proteins move between the producer and target cells^19^,^20^,^21^. Very recently, Inaba *et al*.^22^ described for the first time microtubule-based nanotubes in *Drosophila* testis that resemble TNTs previously described in mammalian cells, that are neither filopodia nor cytonemes. They proposed that these structures contribute to short-range signalling in *Drosophila* niche-stem-cell.

Insects are well-known vectors of a variety of pathogens including viruses, bacteria, protozoa and nematodes^23^. Although insect-borne viral diseases have been a threat to humans since recorded history, insect-virus interactions and mechanisms of insect antiviral immunity remain poorly characterized^24^. The discovery of RNA interference (RNAi) as the major antiviral immune mechanism in invertebrates^25^,^26^,^27^,^28^ has opened new avenues to understand insect immunity. RNAi refers to sequence-specific RNA-dependent silencing mechanisms^29^,^30^ that regulate various processes such as gene expression^31^, epigenetic modifications^32^ and defence against pathogens^33^. Antiviral RNAi is naturally triggered by virus-derived double-stranded RNA (dsRNA) molecules. These long viral dsRNA molecules prompt the small-interfering RNA (siRNA) pathway^29^, silencing both viral dsRNA replicative intermediates as well as viral genomes^34^,^35^,^36^.

The RNAi mechanism is described as either cell-autonomous or non-cell-autonomous^29^,^37^. In cell-autonomous RNAi, the silencing process is limited to the cell in which the dsRNA is introduced or expressed. In non-cell-autonomous RNAi, the interfering effect occurs in cells distinct from those in which the dsRNA was produced. Non-cell-autonomous RNAi presumes that a silencing signal is transported from one cell to another *via* an unknown mechanism to establish antiviral systemic immunity^38^,^39^.

Because of their role in cell-cell communication, we investigated whether membrane-nanotubes could be one of the mediators that connect *Drosophila* cells in order to establish a systemic RNAi-mediated antiviral immune response. We describe the presence of nanotube-like structures in different *Drosophila* cell types. These nanotubes were associated with components of the RNAi system including Argonaute 2, dsRNA, and CG4572^39^. They increased specifically during viral infection and seem to support the transport of Argonaute 2 protein between infected and non-infected cells. We postulate that the spread of the silencing signal in insects could rely, among other cellular mechanisms, on nanotube-like structures forming intercellular connections.

## Results

### *Drosophila* cells are connected to neighbouring cells by nanotube-like structures

**Figure 1:**
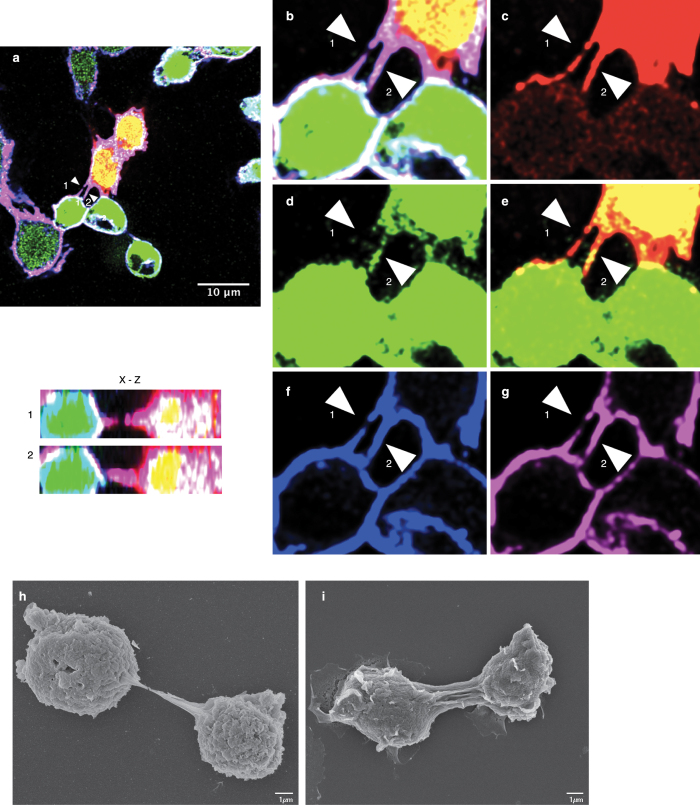
Nanotube-like structures are present in *Drosophila* cells. Stable cell lines expressing eGFP or dsRed under the control of an actin promoter were mixed at a 1:1 ratio, grown overnight and examined by confocal microscopy (**a–g**). Note that images have been voluntarily saturated to better visualize the nanotube-like structures. (**a**) Merged image of eGFP and dsRed cells stained for tubulin and F-actin. Zoom of (**a**) is depicted in (**b**) to better visualize the structures indicated by arrows 1 and 2. (**c**) dsRed positive cells. (**d**) eGFP positive cells. Cells were stained for tubulin in blue (**f**) and F-actin using Phalloidin 647 Alexa-Fluor (**g**). The inset in (**a**) depicts the corresponding (x–z) section through the marked nanotube-like structures (arrow). Arrows indicate projections between cells and bars represent 10 μm (**a**) and 1 μm (**h**–**i**). Scanning electron microscopy of S2 cells showing projections between cells (**h**,**i**).

To test for the presence of membranous connections or nanotube-like structures between cells, we established two stable *Drosophila* S2 cell lines: one expressing dsRed and the other eGFP, each under the control of an actin promoter. This allowed us to distinguish cell-cell connectors from remnants of incomplete cytokinesis events. Cells were mixed 1:1, adhered overnight on glass coverslips, fixed and analysed by confocal microscopy. Membrane projections connecting cells were readily observed (Fig. 1a–g, merge Fig. 1a). The membrane projections observed between both cell types contained tubulin (Fig. 1f) as well as F-actin, as evidenced by positive staining with fluorophore-conjugated Phalloidin (Fig. 1g). Moreover, they were not attached to the substratum (x-z section of structures 1 and 2, arrows). Together, these features are indicative of membrane nanotube-like structures^11^,^22^,^40^. Similar membrane projections were identified in another *Drosophila* cell line, Kc167 (Supplementary Fig. S1), suggesting that nanotube-like structures may be a general feature in *Drosophila*.

To investigate the structure of these tubes, and to further confirm the confocal results, we performed scanning electron microscopy (SEM) and correlative microscopy on S2 cells (Supplementary Fig. S2). SEM revealed the presence of projections connecting neighbouring cells (Fig. 1h,i) as single structure (Fig. 1h) or as multiple nanotube-like connections (Fig. 1i). Correlative microscopy (Supplementary Fig. S2) indicated that these connections had the same features as nanotube-like structures observed by confocal microscopy, including non-adherence and the presence of F-actin^21^. The average diameter of the nanotube-like structure was 250 nm (n = 12), in agreement with the diameter published for TNTs^2^,^5^.

### Virus-infected cells show more abundant nanotube-like structures

**Figure 2:**
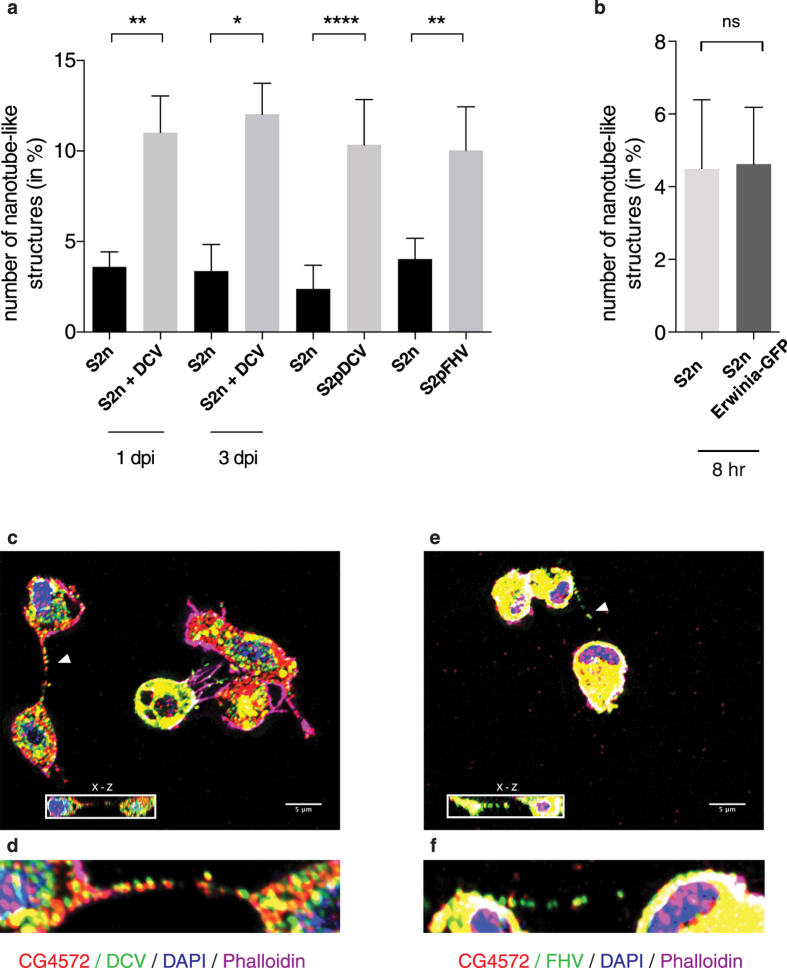
Nanotube-like structures are more abundant during viral infection. (**a,b**) Non-infected S2 cells (S2n), S2 cells during acute DCV infection, S2 cells persistently infected with either DCV (S2pDCV), or with FHV (S2pFHV), and S2 cells infected with bacteria (S2n Erwinia-GFP) were plated on glass coverslips overnight as described in Materials and Methods. Cells were stained for DAPI and Phalloidin, and nanotube-like structures were counted in at least 1,000 cells per treatment group. Error bars indicate standard deviation of the mean of three independent experiments and three biological replicates per experiment. **p ≤ 0.0079. *p = 0.0159. ****p < 0.0001. ns: non-significant. All statistical analyses were performed with Prism 6 software, using a non-parametric Mann-Whitney test. (**c**–**f**) Immunofluorescence during acute infection of S2 cells with DCV (**c**,**d**) or FHV (**e**,**f**). Cells were stained for CG4572 and viral capsid. DAPI and Phalloidin 647 Alexa-Fluor were used to mark nuclei and F-actin, respectively. The insets in (**c**,**e**) depict the corresponding (x–z) section through the marked nanotube-like structure (arrow). Higher magnification images of nanotube-like structure (arrow), viral proteins and CG4572 are shown in (**d**,**f**).

To explore a possible role for nanotube-like structures in antiviral immunity, we then tested whether the abundance of tubes changed in relation to the infection status of the cell. Non-infected cells (S2n), S2 cells during *Drosophila* C virus (DCV) acute infection, cells persistently infected (100% of cells infected) with either DCV (S2pDCV) or flock house virus (FHV) (S2pFHV) were adhered on coverslips for 12 hours and at least 1,000 cells/condition were examined by confocal microscopy as above. There was a significant increase in the number of nanotube-like structure connections during acute infection at day 1 post infection (44% of cells infected by DCV immunostaining) and at day 3 post infection (100% cells infected by DCV immunostaining), as well as in persistently infected cells with connections observed in 10.54% of S2p cells but in only 3% S2n cells (Fig. 2a). However, nanotube-like structure formation could be a consequence of stress due to infection, rather than a means of cell-cell communication during viral infection. Therefore, we counted nanotube-like structures during infection of S2 cells with a bacterium *Erwinia carotovora* expressing GFP. As shown in Fig. 2b, after 8 hours of bacterial infection, bacteria-infected cells displayed as many tubular structures as non-infected cells (4.08% and 4.57% respectively, n > 600). We then checked for the presence of viral proteins associated with the nanotube-like structures of infected cells using polyclonal antibodies generated against viral particles. Readily detectable levels of DCV (Fig. 2c,d) and FHV (Fig. 2e,f) capsid protein were present in those structures together with CG4572, a *Drosophila* protein that is involved in the spread of the RNAi signal *in vivo*^39^, raising the possibility that nanotube-like structures could also be a mechanism for cell-cell spread of virus infection, as was described for TNTs^6^,^7^,^8^,^9^,^10^.

### The RNAi machinery localizes along nanotube-like structures

**Figure 3:**
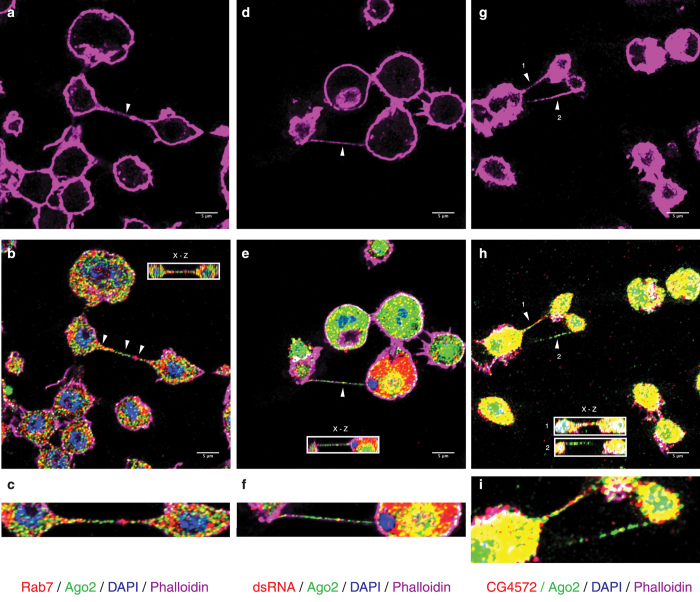
The RNAi machinery localizes along nanotube-like structures. Immunofluorescence and confocal microscopy in S2 cells. Cells were stained for F-actin using Phalloidin 647 Alexa-Fluor (**a**,**d**,**g**). Rab7 and Ago2 (**b**,**c**), dsRNA and Ago2 (**e**,**f**) and CG4572 protein and Ago2 (**h**,**i**) were detected along nanotube-like structures. DAPI is used to mark nuclei. The insets in (**b**,**e**,**h**) depict the corresponding (x-z) section through the marked nanotube-like structure (arrow). Higher magnification images of nanotube-like structures (arrow) and RNAi proteins are shown in (**c**,**f**,**i**).

To determine if the nanotube-like structures participate in RNAi signalling, we looked for the presence of RNAi markers in association with these nanotubes in non-infected (Fig. 3a–c) and FHV infected (Fig. 3d–i) S2 cells. We also looked for late endosomal vesicles shown to be essential for an effective RNAi response^41^,^42^,^43^,^44^. Rab7 (Fig. 3b,c), a marker for late endosomes, and Argonaute 2 (Ago2), the main actor of the antiviral RNAi response^45^, were each detected as dots, along nanotube-like structures and could be observed localizing together down the same tubule (Fig. 3b, arrowsand c). Following infection with FHV, Ago2, dsRNA (Fig. 3e,f) and CG4572 (Fig. 3h,i), also localized along nanotube-like structures. These data support the hypothesis that nanotube-like structures play a role in cell-cell communication during the RNAi response.

### Argonaute 2 localises inside nanotube-like structures and is transferred between cells

**Figure 4:**
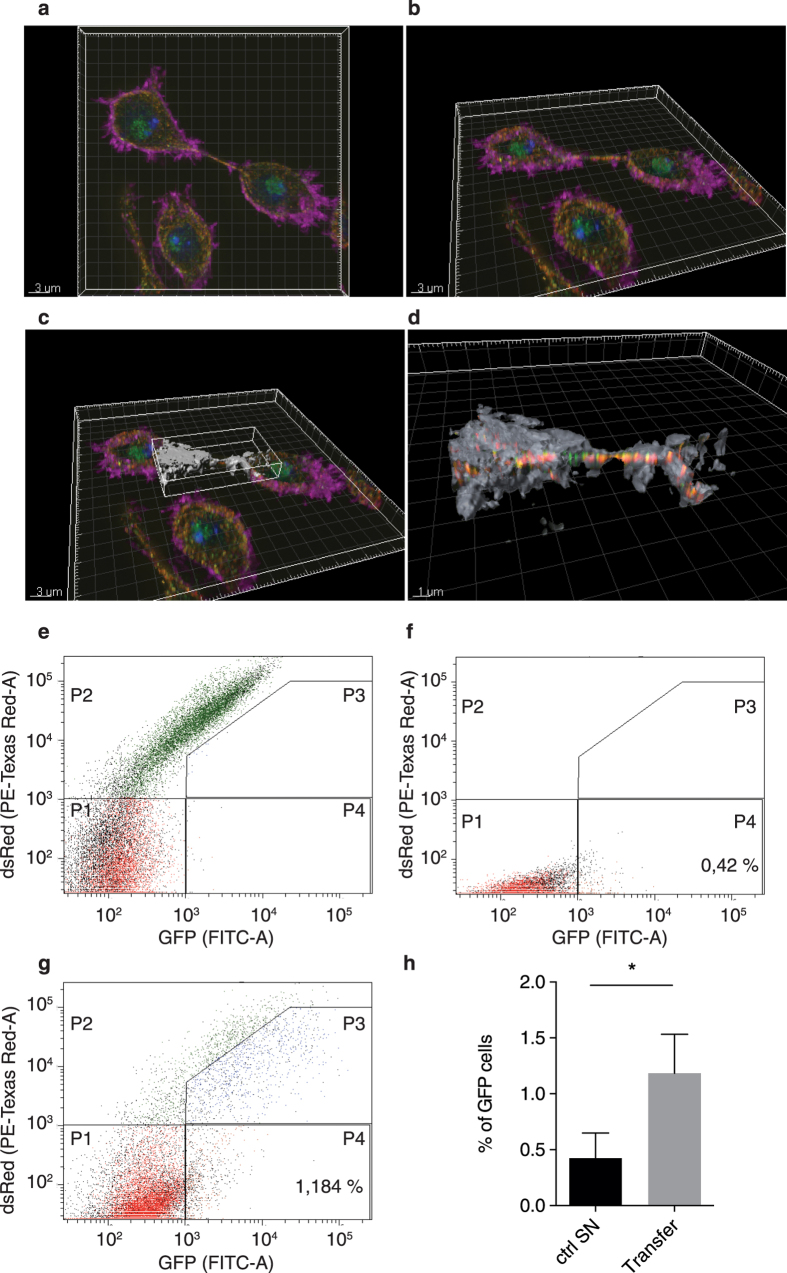
Ago2 localises inside nanotube-like structures and is transferred between cells. *Drosophila* S2 cells were stained for F-actin using Phalloidin 647 Alexa-Fluor, Ago2 in green and Tubulin in red. DAPI was used to stain the nucleus. SR-SIM acquisition in a plan view (**a**) or seen represented as 3D view with an image rotation (**b**). A surface (in grey) was defined following the Phalloidin staining (**c**) and a zoom was performed to only visualize the proteins that localize beneath the surface (proteins outside the surface are not visible in this representation) (**d**). (**e**) S2n cells were mixed 1:1 with S2R+ dsRed cells and gates were set. P1 quadrant shows double negative cells dsRed−GFP−, P2 quadrant shows dsRed+ GFP− cells, P3 quadrant shows double positive cells dsRed+ GFP+, P4 quadrant shows dsRed-GFP+ cells. (**f**) Analysis of S2n cells plated with supernatant of S2R+ dsRed transfected with Ago2-GFP. (**g**) Analysis of S2n cells mixed 1:1 with S2R+ dsRed cells transfected with Ago2-GFP. (**h**) Statistical analysis of the percentage of S2n cells that became GFP+ (P4 quadrant) when plated with the supernatant of transfected cells (0.42%) (**f**) or mixed with the transfected cells (1.184%) (**g**). Error bars indicate standard deviation of the mean of three independent experiments and two biological replicates per experiment. p = 0.0179. All statistical analyses were performed with Prism 6 software, using a non-parametric Mann-Whitney test.

Ago2 could be present outside nanotube-like structures, supporting the transfer of surface-associated cargo from cell to cell, or could be inside the structures, allowing the exchange of molecules through cytoplasmic connection between two cells. To address the sub-localisation of Ago2 in the nanotube-like structure with more resolution and accuracy, we performed super resolution structured illumination microscopy (SR-SIM) 3D instead of confocal microscopy. Figure 4a,b show the acquisition of the signal for Ago2, F-actin and tubulin in S2 cells using SR-SIM. Figure 4c shows the definition of the surface area with F-actin signal that was generated around the nanotube-like structure. Using the surface tool of the Imaris software along the Phalloidin staining, Ago2 and tubulin clearly appeared to be contained inside the structure (Fig. 4d).

Once we established that Ago2 and tubulin were inside the tubules, we analysed whether Ago2 from infected cells could be found in non-infected cells following co-culture (Fig. 4e–h). To do this, we mixed S2n cells with persistently infected S2R+ dsRed cells transfected with Ago2-GFP. After three days of incubation, FACS analysis revealed that S2n cells became GFP+ in a significant manner (p = 0.0179) due to Ago2-GFP transfer between cells (Fig. 4g,h). On the other hand, S2n cells incubated for three days with a three days old supernatant of S2R+ dsRed cells transfected with Ago2-GFP remained GFP negative (Fig. 4f), discarding the possibility of circulation of Ago2-GFP through extracellular media. Although we cannot exclude that Ago2-GFP is being transferred between infected and non-infected cells through other type of intercellular connections, the low level of S2nGFP+ cells is in agreement with the frequency of detection of nanotube-like structures.

## Discussion

Intercellular communication must be highly selective and tightly regulated as it is essential for the survival of any organism. In recent years, cell-cell connections between animal cells, including tunnelling nanotubes and filopodia, were discovered and thought to facilitate trafficking of cytoplasmic material^5^, transmission of calcium signals^46^ or pathogens^10^,^11^, and recently short-range signalling in *Drosophila* niche-stem-cell^22^. Here, we identify nanotube-like structures in *Drosophila* cells in the context of a viral infection, and we provide evidence that they may also function in cell-cell communication in response to infection. *Drosophila* cells show nanotube-like structures that are positive for F-actin as well as tubulin staining and are non-adherent. The nanotube-like structures contain components of the RNAi system, including Ago2, dsRNA, Rab7, and CG4572^39^. In addition, they increased in abundance during virus infection and contained viral capsid proteins, some of which localized together with components of the RNAi machinery. Together, this suggests a participation of nanotube-like structures in establishing systemic RNAi anti-viral immunity in *Drosophila*.

In 2011, Lopez-Montero *et al*.^47^ observed that mosquito cells infected with an arbovirus (Bunyamwera virus) developed a complex network of filopodium-like bridges and proposed they could serve for virus propagation, but most likely to spread protective signals between cells. Our results suggest that nanotube-like structures could be one of the mechanisms by which antiviral RNAi machinery is transported from a donor to an acceptor cell to trigger intracellular antiviral immunity in the latter. The presence of viral capsid protein associated with the nanotube-like structures raises the possibility that they may also be a means of cell-to-cell spread of virus, as proposed by Lopez-Montera^47^ and similar to HIV^10^. At present, it remains unfeasible to investigate these hypotheses, as that would require the disruption of nanotube-like structures during viral infection. One of the only methods described to disrupt nanotubes is to inhibit actin polymerization by latrunculin A and B^5^. However, the disruption of actin polymerization strongly affects intra- and intercellular movement of viruses such as entry and budding^48^; thereby precluding its utilization and leaving us, for the time being, at the speculative level.

The observation that nanotube-like structures are present in *Drosophila* S2 cells reveals a powerful model to study their biogenesis and mechanisms of cell-cell communication. For example, while TNTs are widely thought to play a role in cell-cell communication, it remains controversial if they are open- or closed-ended^40^. We found evidence that Ago2 is present within the tubules (and not at their surface) and our data suggest that Ago2-GFP is transferred from infected to non-infected cells *via* cell-cell communication, supporting the idea that nanotube-like structures could be one of the means of transport of the RNAi machinery. Additionally, as *Drosophila* is a genetically tractable small animal susceptible to a number of natural virus infections, approaches could be developed to explore the relevance of nanotube-like structures for antiviral immunity *in vivo*, a question still pending in biology.

## Methods

### Cell culture

Non-infected (S2n) and persistently infected (S2R+) *Drosophila* S2 cells (Schneider, 1972, invitrogen) were cultured at 25 °C in Schneider’s *Drosophila* medium (invitrogen) supplemented with 10% heat inactivated fetal bovine serum (FBS, invitrogen), 2 mM glutamine (invitrogen), 100 U/mL penicillin and 100 μg/mL streptomycin (invitrogen).

### Transfection and stable cell line establishment

1 × 10^7^ cells were cotransfected with pAc5.1B-eGFP (plasmid #21181 from AddGene) or with pAc-dsRed (kindly provided by Dr. F. Coumailleau) and pCoBlast vector (invitrogen) in a 19:1 ratio using Effectene reagent (Qiagen). Five days later, cells were selected by replacing Schneider’s complete *Drosophila* media with fresh medium supplemented with Blasticidin (25 μg.mL^−1^, Euromedex). By 14 days later, 98% of cells expressed eGFP or dsRed protein.

### Immunofluorescence

*Drosophila* S2 cells were cultured overnight on coverslips at a concentration of 5 × 10^5^ cells/mL in complete Schneider’s *Drosophila* medium, as described above. Cells were then fixed in paraformaldehyde diluted in PBS to 4% (PFA, Alfa Aesar) during 15 minutes (min) and washed two times in PBS for 5 min. Following the fixation step, permeabilization was done in PBS 0.1% Triton X-100 (PBS-Triton) for 5 min three times. Cells were then incubated with primary antibodies in PBS-Triton supplemented with 3% FBS for at least 1 hour (hr) at RT (rabbit αFHV 1/500, rabbit αDCV 1/500, and mouse αCG4572 1/500 are three home-made antibodies, mouse anti-dsRNA αK1 1/1,000 Scicons, rabbit αAgo2 1/500 Abcam or mouse αAgo2 1/100 kindly provided by Pr. Siomi and rabbit αRab7 1/2,000 kindly provided by Pr. Nakamura). After three washes in PBS-Triton, cells were incubated with secondary antibody (Alexa-Fluor 1/1,000, invitrogen), DAPI (1/10,000, life technologies) and Phalloidin-647 (Alexa-Fluor 1/200, invitrogen) diluted in PBS-Triton with 3% FBS for 1 hr at RT. Cells were washed two times in PBS-Triton for 5 min, once in PBS and finally mounted on glass slide with Fluoromount G (eBioscience) and imaged with a confocal microscope LSM 700 inverted (Zeiss) at a 63X magnification oil immersion lens. Brightness and color balance in some images have been changed only in order to increase visibility of nanotube-like structures. Confocal stacks were deconvolved in Huygens Professional software (SVI).

### Virus and bacteria infections

*Drosophila* S2n cells were cultured on glass coverslip as previously described, during an overnight acute infection for DCV (1 M.O.I.) or FHV (1 M.O.I.). The next day or three days later, cells where harvested, washed, fixed, and immunofluorescence was performed. For persistently infected cells with DCV or FHV, cells were grown as described and IF was performed. For bacteria infections, *Drosophila* S2 cells were washed twice (5 min at 500 rpm) with Schneider’s *Drosophila* medium without antibiotics and plated on glass-coverslips at 5 × 10^5^ per well o/n at 25 °C. Bacterium *Erwinia carotovora carotovora 15* (Ecc15-GFP) (kindly provided by B. Lemaitre)^49^, was grown o/n at 29 °C in LB supplemented with Rifampicin. The next day, bacteria were washed twice in PBS (5 min at 1,000 rpm) and incubated on the glass-coverslip plated S2 cells at 250,000 bacteria/well (OD_600_ = 0.3 = 1 × 10^8^ cell/mL) for 45 min at 25 °C. When adding bacteria on cells, a short spin (1 min at 3,000 rpm) was performed for bacteria to sit on the cells. After 45 min of incubation, the bacteria-containing media was removed and replace with fresh Schneider’s *Drosophila* medium without antibiotics. *Drosophila* S2n cells infected with Ecc15-GFP were harvested at 8 hours post infection, fixed in PFA 4%, stained for DAPI and Phalloidin, and nanotube-like structures were counted as described above. The values represent the mean and SD of three independent experiment and three biological replicate per experiment. **p ≤ 0.0079. *p = 0.0159. ****p < 0.0001. Statistical analysis was performed with Prism 6 software using a non-parametric Mann-Whitney test.

### Correlative microscopy: CLEM-SEM

S2 cells were first imaged by fluorescence microscopy on an alphanumeric coded, grid-patterned glass (MatTek dishes). Samples were fixed in 2.5% glutaraldehyde in 0.1 M cacodylate buffer (pH 7.2) o/n at 4 °C, then washed in 0.2 M cacodylate buffer (pH 7.2), postfixed for 1 hr in 1% osmium and rinsed with distilled water. Cells were dehydrated through a graded ethanol series followed by critical point drying with CO_2_. Dried specimens were gold/palladium sputter-coated with a gun ionic evaporator PEC 682. The samples are imaged in a JEOL JSM 6700 F field emission scanning electron microscope operating at 5 kV.

### Super-resolution structured illumination microscopy (SR-SIM)

S2 cells were cultured o/n on a glass coverslips as previously described. IF was performed as described, using rabbit αAgo2 1/500 Abcam and mouse αTubulin 1/5,000 Sigma as primary antibodies. Cells were then incubated with secondary antibody (Alexa-Fluor 1/1,000, invitrogen), DAPI (1/10,000, life technologies) and Phalloidin-647 (Alexa-Fluor 1/200, invitrogen) and mounted on glass slides with SlowFade gold (ThermoFisher). SR-SIM was performed on a Zeiss LSM780 Elyra PS1 (Carl Zeiss, Germany) using 63x/1.4 oil Pln Apo objective. Three angles of the excitation grid with five phases each were acquired for each channel and each z-plane. SIM images were processed with ZEN software and then aligned with ZEN using 100 nm tetraspeck beads embedded in the same conditions as the sample. SIMcheck plugin in imageJ^50^ was used to analyze the acquisition and the processing in order to optimize between resolution/signal to noise ratio/artifacts.

Following the SR-SIM and using Imaris software, a surface defined by the Phalloidin signal was generated around the nanotube-like structure. Only Ago2 and Tubulin contained inside this surface could then be observed.

### Ago2 transfer experiment

10 × 10^6^ S2R+ dsRed cells were plated and transfected with Ago2-GFP plasmid using Effectene reagent (Qiagen), following manufacturer’s recommendations. The next day, cells were washed. Three days after transfection, supernatant of cells was collected, spanned at 1,200 rpm for 5 min and filtered with a 0.22 μm filter, to eliminate the presence of some leftover cells, and plated over S2n cells. The same day, S2R+ dsRed cells transfected with Ago2-GFP were collected and sorted for dsRed+ GFP+ on a MoFlo ASRIOS (Beckman Coulter). Sorted cells were plated in 1:1 ratio together with S2n cells, incubated for 3 days, and analysed on a FACS Diva. The values represent the mean and SD of three independent experiment. *p = 0.0179. Statistical analysis was performed with Prism 6 software using a non-parametric Mann-Whitney test.

## Additional Information

**How to cite this article**: Karlikow, M. *et al. Drosophila* cells use nanotube-like structures to transfer dsRNA and RNAi machinery between cells. *Sci. Rep.* **6**, 27085; doi: 10.1038/srep27085 (2016).

## Supplementary Material

Supplementary Information
