# Supplementary material for: Drosophila cells use nanotube-like structures to transfer dsRNA and RNAi machinery between cells
Source: Sci Rep. 2016 Jun 3;6:27085. doi: 10.1038/srep27085 (PMC4891776; doi:10.1038/srep27085)
Supplement: Supplementary Information [file srep27085-s1.pdf]

Drosophila cells use nanotube-like structures to transfer dsRNA and RNAi machinery between cells

Margot Karlikow[1,5], Bertsy Goic[1], Vanesa Mongelli[1], Audrey Salles[2], Christine Schmitt[3], Isabelle Bonne[3], Chiara Zurzolo[4], Maria-Carla Saleh[1]*

1- Institut Pasteur, Viruses and RNA interference, CNRS URM 3569, 75724 Paris Cedex 15, France.
2- Institut Pasteur, Imagopole, Citech, 75724 Paris Cedex 15, France.
3- Institut Pasteur, Platform of Ultra-structural microscopy, 75724 Paris Cedex 15, France.
4- Institut Pasteur, Membrane traffic and pathogenesis, 75724 Paris Cedex 15, France.
5- Sorbonne Universités, UPMC Université Paris VI, IFD, 4 Place Jussieu 75252 Paris Cedex 05, France.

Correspondence: carla.saleh@pasteur.fr (M.C.S.)

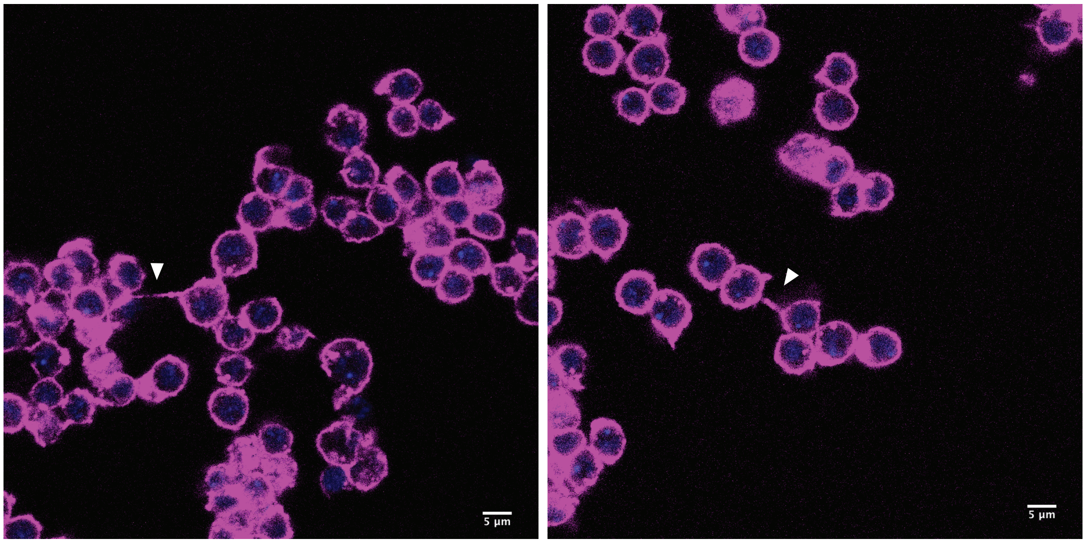

DAPI / Phalloidin

Supplementary Figure S1:

Nanotube-like structures are present in Drosophila Kc167 cells. Immunofluorescence and confocal microscopy. Cells were stained for F-actin using Phalloidin 647 Alexa-Fluor to show membrane continuity between connected cells. DAPI is used to mark nuclei.

Supplementary Figure S1
Karlikow *et al.*

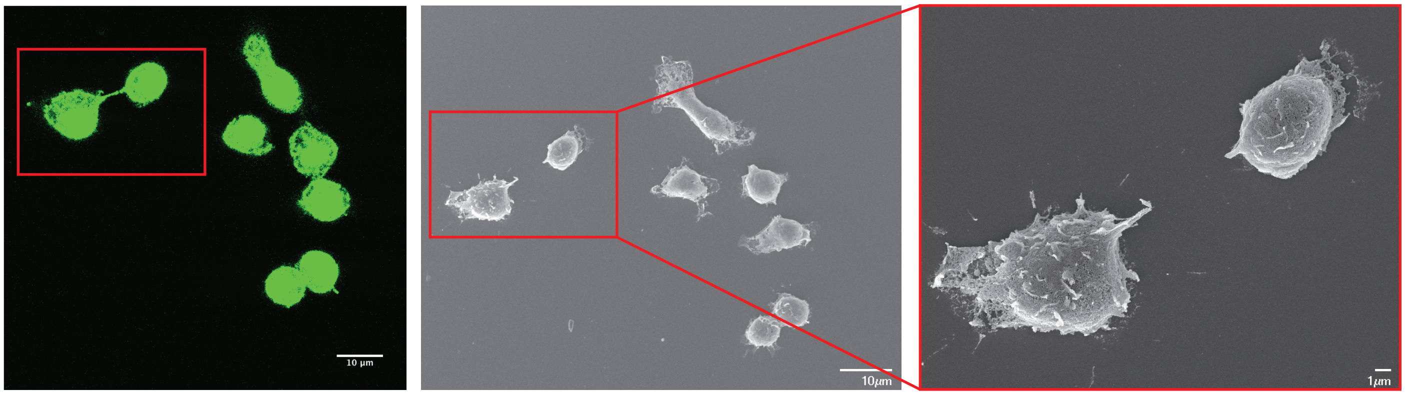

Supplementary Figure S2:

Correlative microscopy of nanotube-like structures. S2 cells were grown overnight on an alphanumeric coded, grid-patterned glass and imaged for Ago2-along nanotube-like structures in confocal microscopy (red square). The alphanumeric code allowed localization, and scanning electron microscopy was performed on the exact same cells.

Supplementary Figure S2
Karlikow *et al.*
